# Comparison of Efficacy between 120° and 180° Schlemm’s Canal Incision Microhook Ab Interno Trabeculotomy

**DOI:** 10.3390/jcm10143181

**Published:** 2021-07-19

**Authors:** Naoki Okada, Kazuyuki Hirooka, Hiromitsu Onoe, Yumiko Murakami, Hideaki Okumichi, Yoshiaki Kiuchi

**Affiliations:** Department of Ophthalmology and Visual Science, Hiroshima University, Hiroshima 734-8551, Japan; naokimed@hiroshima-u.ac.jp (N.O.); onoehir@hiroshima-u.ac.jp (H.O.); yumiko00@hiroshima-u.ac.jp (Y.M.); okumic@hiroshima-u.ac.jp (H.O.); yoshiaki@hiroshima-u.ac.jp (Y.K.)

**Keywords:** ab interno trabeculotomy, intraocular pressure, glaucoma, incision in the Schlemm’s canal in degrees, post-surgical complication

## Abstract

We compared surgical outcomes in patients with either primary open-angle glaucoma or exfoliation glaucoma after undergoing combined phacoemulsification with either a 120° or 180° incision during a Schlemm’s canal microhook ab interno trabeculotomy (μLOT-Phaco). This retrospective comparative case series examined 52 μLOT-Phaco eyes that underwent surgery between September 2017 and December 2020. Surgical qualified success was defined as an intraocular pressure (IOP) of ≤20 mmHg, ≥20% IOP reduction with IOP-lowering medications, and no additional glaucoma surgery. Success rates were evaluated by Kaplan-Meier survival analysis. The number of postoperative IOP-lowering medications and occurrence of complications were also assessed. Mean preoperative IOP in the 120° group was 16.9 ± 7.6 mmHg, which significantly decreased to 10.9 ± 2.7 mmHg (*p* < 0.01) and 11.1 ± 3.1 mmHg (*p* = 0.01) at 12 and 24 months, respectively. The mean number of preoperative IOP-lowering medications significantly decreased from 2.8 ± 1.4 to 1.4 ± 1.4 (*p* < 0.01) at 24 months. Mean preoperative IOP in the 180° group was 17.1 ± 7.0 mmHg, which significantly decreased to 12.1 ± 3.2 mmHg (*p* = 0.02) and 12.9 ± 1.4 mmHg (*p* = 0.01) at 12 and 24 months, respectively. The mean number of preoperative IOP-lowering medications significantly decreased from 2.9 ± 1.2 to 1.4 ± 1.5 (*p* < 0.01) at 24 months. The probability of qualified success at 24 months in the 120° and 180° groups was 50.4% and 54.6%, respectively (*p* = 0.58). There was no difference observed for hyphema formation or IOP spikes. Surgical outcomes were not significantly different between the 120° and 180° incisions in Schlemm’s canal.

## 1. Introduction

Worldwide, glaucoma is the second most common cause of blindness [1]. Elevated intraocular pressure (IOP) has been reported by several studies to be an important risk factor for glaucoma and disease progression [2,3]. First-line therapy involves topical medications, which have a demonstrated and proven record of efficacy in all adult glaucoma stages. Incisional surgery is often considered when topical medications are not able to adequately reduce the IOP. New devices introduced over the last few years include minimally invasive glaucoma surgery (MIGS), which has generated great interest within the field. The greatest resistance for the aqueous humor outflow has been shown to be within the trabecular meshwork, with glaucomatous eyes exhibiting an even greater resistance in this region [4]. Devices and techniques, such as the trabectome, microhook, and Kahook Dual Blade, have been employed as ways for reducing the resistance of the trabecular meshwork.

While using a gonio lens, Tanito et al. [5] performed an ab interno trabeculotomy procedure, which utilized an original microhook (Inami & Co., Ltd., Tokyo, Japan), in order to incise the internal wall of the trabecular meshwork. In these original studies, Tanito et al. [5,6] utilized a 240° incision of the trabecular meshwork. In our own recent study, we found that after combined phacoemulsification with either a microhook or a Kahook Dual Blade ab interno trabeculotomy, there was a significant increase in the corneal higher-order aberrations (HOAs) [7]. After examining the extent of the incision in the Schlemm’s canal in degrees (EIS) (which included both a 120° and 180° group), we determined that the risk factor associated with the increased corneal HOAs was the EIS in the 180° Schlemm’s canal opening group [7]. Based on these previously reported findings, this suggests that both the efficacy and safety of the 120° or 180° of the EIS should be investigated in a microhook ab interno trabeculotomy (μLOT).

Therefore, the purpose of our current study was to examine the efficacy and safety of both the 120° and the 180° incisions in phacoemulsification cataract extraction with μLOT (μLOT-Phaco).

## 2. Materials and Methods

This study evaluated data obtained from patients who underwent μLOT-Phaco between September 2017 and December 2020 at Hiroshima University Hospital. The study protocol was approved by the Institutional Review Board of Hiroshima University. All subjects provided written informed consent in accordance with the principles outlined in the Declaration of Helsinki, in addition to the standard consent for surgery.

Although patients with primary open-angle glaucoma (POAG) and exfoliation glaucoma were included in the study, any patient with a history of previous ocular surgery was excluded. In addition, all subjects had to have a minimum of 3 months of postoperative follow-up in order to be included in the analysis. When both eyes were treated in the patient, only the first eye treated was used for the analysis.

A 2.8-mm incision was created in the temporal position of the cornea. Removal of the nucleus during all procedures was performed using a standard phacoemulsification technique: Whitestar Signature Pro (Johnson & Johnson, New Brunswick, NJ, USA). All of the intraocular lens (IOLs) (PCB00V, Johnson & Johnson; XY1, HOYA, Tokyo, Japan) were implanted in the capsular bag. The μLOT surgical technique has been previously described [8]. In brief, after the cataract surgery, μLOT was performed under direct gonioscopy, with the microhook inserted into the anterior chamber through the corneal incision. Subsequently, after insertion of the tip of the microhook into Schlemm’s canal, it was then moved circumferentially in order to incise the inner wall of the Schlemm’s canal and the trabecular meshwork at 4 clock hours (nasal quadrant: 120°) or at 6 clock hours (inferior and nasal bisection: 180°) (Video, Supplemental Digital Content, Video S1). All decisions regarding the degree of the incision in Schlemm’s canal were made by the surgeon performing the procedure. A 27-gauge cannula was used to hydrate the incisions after the removal of the ophthalmic viscosurgical device. Each individual surgeon was responsible for the postoperative administration of anti-inflammatory, anti-infective, and miotic therapies. Furthermore, the restarting of the IOP-lowering medications was performed in accordance with the judgment of the surgeon.

All statistical analyses were conducted using JMP software version 15 (SAS Inc., Cary, NC, USA). The Student’s *t*-test for continuous variables and a chi-square test for categorical variables were used to compare the clinical characteristics between the 120° and 180° incision groups. A chi-square test was also used to compare the postoperative complications. In addition, mean IOP and mean number of IOP-lowering medications, along with their reductions from baseline, were determined at each time point by group and compared between groups. When a combination of IOP-lowering medications was used, this was counted as two drugs. This study also performed a Kaplan-Meier survival analysis, with a drop of the IOP of at least 20%, an IOP ≤ 20 mmHg with (qualified success) or without (complete success) administration of IOP-lowering medications, and no additional glaucoma surgery all defined as being a success. Surgical success was achieved if, after ≥3 months of follow-up because of the occurrence of postoperative IOP fluctuations after trabeculotomy [9]. Continuous data are presented as the mean ± standard deviation. *p* values < 0.05 were considered to indicate statistical significance.

## 3. Results

A total of 52 consecutive eyes from 52 patients with open-angle glaucoma (POAG: 34, exfoliation glaucoma: 18) were included in this study. Table 1 presents the clinical characteristics of the enrolled participants. There were no significant differences observed between the 120° and 180° incision groups in terms of age, gender, glaucoma type, preoperative IOP, and the number of glaucoma eye drops.

**Figure 1:**
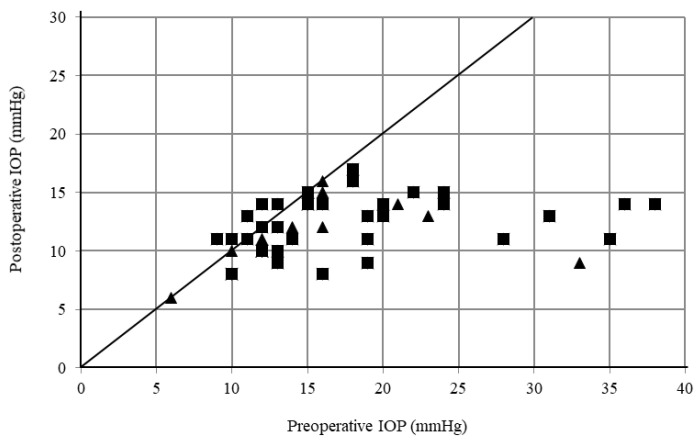
Scattergram of preoperative versus postoperative intraocular pressure (IOP). Triangle: 120° incision group, Square: 180° incision group.

Table 2 shows the IOP and changes from baseline at each time point for each group. The mean IOP in the 120° incision group was 16.9 ± 7.6 mmHg at baseline, while it was 12.5 ± 2.7 mmHg (*n* = 25; *p* = 0.01), 10.9 ± 2.7 mmHg (*n* = 14; *p* < 0.01), 11.2 ± 3.0 mmHg (*n* = 13; *p* < 0.01), and 11.1 ± 3.1 mmHg (*n* = 10; *p* = 0.01) at 6, 12, 18, and 24 months, respectively. In the 180° incision group, the IOP was 17.1 ± 7.0 mmHg at baseline, while it was 12.9 ± 2.4 mmHg (*n* = 22; *p* = 0.045), 12.1 ± 3.2 mmHg (*n* = 21; *p* = 0.02), 12.6 ± 2.7 mmHg (*n* = 16; *p* = 0.02), and 12.9 ± 1.4 mmHg (*n* = 13: *p* = 0.01) at 6, 12, 18, and 24 months, respectively. Figure 1 shows pre-operative and postoperative (final visit) IOPs for all patients in the study.

**Figure 2:**
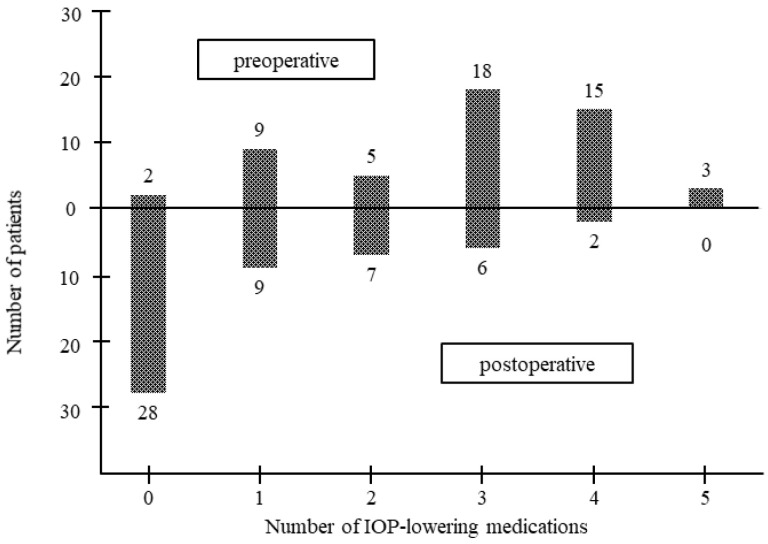
Bar graph showing the number of preoperative and postoperative medications.

Table 3 shows the mean number of IOP-lowering medications at each time point for each group. The mean number of IOP-lowering medications in the 120° incision group was 2.8 ± 1.4 at baseline, while it was 0.9 ± 1.1 (*p* < 0.01), 1.0 ± 1.3 (*p* < 0.01), 1.1 ± 1.3 (*p* < 0.01), and 1.4 ± 1.4 (*p* < 0.01) at 6, 12, 18, and 24 months, respectively. In the 180° incision group, the mean number of IOP-lowering medications was 2.9 ± 1.2 at baseline, while it was 0.9 ± 1.1 (*p* < 0.01), 0.9 ± 1.1 (*p* < 0.01), 1.2 ± 1.3 (*p* < 0.01), and 1.4 ± 1.5 (*p* < 0.01) at 6, 12, 18, and 24 months, respectively. Figure 2 shows the numbers of pre-operative and postoperative IOP-lowering medications.

**Figure 3:**
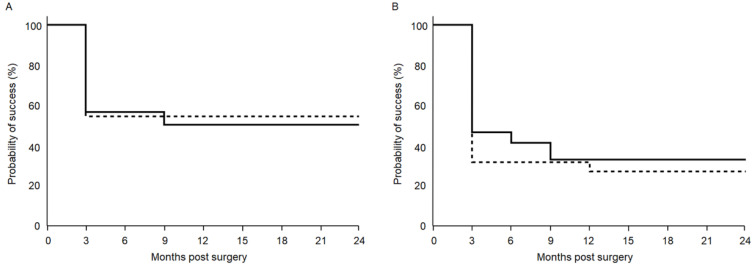
Survival cure analysis success rate of intraocular pressure control after combined phacoemulsification when using either a 120° or 180° incision during Schlemm’s canal microhook ab interno trabeculotomy. Surgery failure was defined as an intraocular pressure drop of < 20%, intraocular pressure > 20 mmHg with (**A**) or without (**B**) intraocular pressure-lowering medication, and additional glaucoma surgery. Solid line: 120° group, Dashed line: 180° group.

Figure 3 shows the Kaplan-Meier survival curves used to compare the surgical outcomes in the 120° and 180° groups. At 12 and 24 months postoperatively, qualified success rates in the 120° and 180° groups were 50.4% and 54.6%, and 50.4% and 54.6%, respectively (*p* = 0.87) (Figure 3A). In contrast, the complete success rates in the 120° and 180° groups were 33.2% and 27.3%, and 33.2% and 27.3%, respectively (*p* = 0.58) (Figure 3B).

Within 1 week after the surgery in the 120° and 180° groups, IOP spikes (an IOP increase of > 30 mmHg) occurred in 2 (6.7%) and 1 (4.6%) eyes, respectively (*p* = 0.75). All of these subsided in conjunction with the administration of IOP-lowering medications. Hyphema was defined as red blood cells accumulated in the anterior chamber of the eye [10]. In the 120° and 180° groups, hyphema with niveau formation occurred in 5 (16.7%) and 4 (18.2%) eyes, respectively (*p* = 0.89) (Table 4). In the 120° group, the extent of the niveau formation of hyphema was below 2 mm in 1 eye, while it was below 1 mm in 4 eyes, with a mean time of resolution of the hyphema of 7.2 days. In the 180° group, the extent of the niveau formation of hyphema in all eyes was below 1 mm, with a mean time of resolution of the hyphema of 4.3 days. None of the eyes required any additional glaucoma surgery.

## 4. Discussion

The results of the current study showed there was a similar efficacy between the 120° and the 180° incision in the Schlemm’s canal μLOT-Phaco procedures. In addition, there were no significant difference observed for the surgical success rate, mean IOP, IOP reduction rate, and IOP-lowering medications scores between the 120° and 180° incisions in Schlemm’s canal.

Manabe et al. [11] examined increases in the extent of the incision in Schlemm’s canal to ≥150° and reported that this did not affect the reduction in the IOP at 1 year postoperatively. Sato et al. [12] also showed that neither the 180° nor the 360° incision in Schlemm’s canal affected the IOP reduction. Furthermore, Mori et al. [13] reported that there was no significant difference for the 1-year success rate between the 1- and 2-quadrant incisions in Schlemm’s canal when using μLOT. In enucleated human eyes, as compared to results for the 360° incision in the Schlemm’s canal LOT, the 30° and 120° incisions resulted in 42% and 85% reductions in outflow resistance, respectively [14]. Therefore, incisions greater than 120° may not be able to further reduce the IOP in the different types of MIGS. In contrast, the 360° suture trabeculotomy achieved lower IOP values as compared to the 120° metal trabeculotomy [15]. However, in that study, the 120° incisions were created with metal probes. It may be difficult to evaluate surgical outcomes according to the extent of the incision because of the different incision approaches.

A previous study reported finding a positive correlation between the extent of the incision in Schlemm’s canal (ranging from 150 to 320°) and the hyphema score at 1 day postoperatively [11]. Furthermore, another study found that there was a significant difference between the 180° and 360° Schlemm’s canal incisions for the frequency of postoperative hyphema [11]. However, our current study found no significant differences for either the complications noted between the 120° and 180° incisions or for the safety between these two techniques. In addition, Mori et al. [13] recently examined the hyphema formation between 1- and 2-quadrant incisions in Schlemm’s canal groups and reported finding there was no difference between the groups. These results suggest that incisions more than 180° in Schlemm’s canal are likely to cause greater blood reflux from the collector channels, thereby leading to a higher frequency of postoperative hyphema.

Our recent study additionally showed that a more extensive incision (180° vs. 120°) in Schlemm’s canal was a risk factor for potential changes in the corneal HOAs, coma-like, and spherical-like aberrations [7]. Corneal HOAs in the 120° Schlemm’s canal incision group were 0.227 ± 0.128 μm at baseline and 0.251 ± 0.131 μm (*p* = 0.12) at 3 months after surgery [7]. However, corneal HOAs in the 180° Schlemm’s canal incision group were 0.216 ± 0.095 μm at baseline and 0.439 ± 0.223 μm (*p* = 0.001) at 3 months after surgery [7]. These results suggest that increases in the HOAs can subsequently lead to functional visual acuity decreases. In order to definitively confirm these findings, we are presently evaluating the vision-related quality of life that occurs after patients undergo μLOT-Phaco procedure.

We evaluated data obtained from patients who underwent μLOT-Phaco in the current study. However, Chen et al. [16] previously reported an average 13% reduction in IOP and 12% reduction in IOP-lowering medications 1 year after phacoemulsification in patients with POAG. Therefore, the true effect associated with the μLOT procedure is difficult to determine.

In our current study, there were some limitations. First, this was a retrospective study. As a result, the 120° or 180° incisions in the Schlemm’s canal were not randomly assigned to the subjects. Furthermore, outcome data were not specifically collected but rather obtained during our routine clinical practice. Second, this study only evaluated a relatively small number of cases. A small sample size might have led to the non-significant difference between the 120° and 180° incisions in Schlemm’s canal. Therefore, in order to obtain better rigorous comparative evidence and data, a multi-center, randomized, prospective study will need to be undertaken. Recent MIGS procedures have been performed in relatively medically controlled glaucoma patients to reduce medication [17]. Such cases were included in the current study and had a relatively low baseline IOP and IOP reduction in each group. In the current study, all cases of failure had a <20% IOP reduction in all patients. To verify the results in eyes with a higher baseline IOP, a subgroup of cases with a baseline IOP ≥ 21 mmHg was analyzed. The mean IOP in the 120° incision group was reduced from 31.2 ± 6.3 mmHg (*n* = 5) at baseline to 12.0 ± 2.4 mmHg at the final visit. The mean IOP in the 180° incision group was also reduced from 26.5 ± 5.8 mmHg (*n* = 6) at baseline to 13.8 ± 0.8 mmHg at the final visit. These results were comparable to those of the original study population.

In conclusion, the current findings demonstrate that there was no difference in the surgical success between the 120° and 180° incisions in the Schlemm’s canal μLOT-Phaco. However, corneal HOAs, coma-like, and spherical-like aberrations were associated with a more extensive incision in Schlemm’s canal, thereby suggesting that extensive incisions are a risk factor in these procedures. Therefore, the authors strongly recommend the use of a 120° incision during Schlemm’s canal μLOT-Phaco.

## Figures and Tables

**Table 1:** Clinical characteristics.

	120°	180°	*p* Value
No. eyes	30	22	
Age (years)	72.3 ± 10.4	75.4 ± 10.8	0.30
Gender (M/F)	18/12	13/9	0.94
Type of glaucoma			0.82
POAG	20	14	
Exfoliation glaucoma	10	8	
Preoperative IOP (mmHg)	16.9 ± 7.6	17.1 ± 7.0	0.94
No. IOP-lowering medications	2.8 ± 1.4	2.9 ± 1.2	0.89
Mean deviation (dB)	−13.9 ± 1.4	−9.2 ± 1.7	0.03

M; male, F; female, POAG; primary open-angle glaucoma; IOP; intraocular pressure.

**Table 2:** Differences in preoperative and postoperative IOP.

		120°			180°		
	IOP at Each Time Point (mmHg)	Change from Baseline (%)	*p* Value *	IOP at Each Time Point (mmHg)	Change from Baseline (%)	*p* Value *	*p* Value **
Baseline	16.9 ± 7.6 (*n* = 30)			17.1 ± 7.0 (*n* = 22)			0.94
Month 6	12.5 ± 2.7 (*n* = 25)	18.8 ± 26.6	0.01	12.9 ± 2.4 (*n* = 22)	15.3 ± 28.6	0.045	0.62
Month 12	10.9 ± 2.7 (*n* = 14)	29.5 ± 29.7	<0.01	12.1 ± 3.2 (*n* = 21)	24.1 ± 25.4	0.02	0.27
Month 18	11.2 ± 3.0 (*n* = 13)	26.1 ± 31.2	<0.01	12.6 ± 2.7 (*n* = 16)	22.7 ± 29.3	0.02	0.77
Month 24	11.1 ± 3.1 (*n* = 10)	34.2 ± 27.2	0.01	12.9 ± 1.4 (*n* = 13)	23.1 ± 26.4	0.01	0.07

IOP; intraocular pressure; * Calculated using paired *t*-test for IOP between preoperative and postoperative value; ** Calculated using Student’s *t*-test for % changes from baseline between the groups.

**Table 3:** Differences in preoperative and postoperative number of IOP-lowering medication.

	120°		180°		
	Number of Medications at Each Time Point	*p* Value *	Number of Medications at Each Time Point	*p* Value *	*p* Value **
Baseline	2.8 ± 1.4 (*n* = 30)		2.9 ± 1.2 (*n* = 22)		0.89
Month 6	0.9 ± 1.1 (*n* = 25)	<0.01	0.9 ± 1.1 (*n* = 22)	<0.01	0.94
Month 12	1.0 ± 1.3 (*n* = 14)	<0.01	0.9 ± 1.1 (*n* = 21)	<0.01	0.86
Month 18	1.1 ± 1.3 (*n* = 13)	<0.01	1.2 ± 1.3 (*n* = 16)	<0.01	0.78
Month 24	1.4 ± 1.4 (*n* = 10)	<0.01	1.4 ± 1.5 (*n* = 13)	<0.01	>0.99

IOP; intraocular pressure; * Calculated using Wilcoxon signed-ranks test for number of IOP-lowering medication between preoperative and postoperative values; ** Calculated using Mann-Whitney’s U test for postoperative values between the groups.

**Table 4:** Postoperative complications.

	120°	180°	*p* Value
Hyphema with niveau formation	5 (16.7%)	4 (18.2%)	0.89
Transient IOP elevation > 30 mmHg	2 (6.7%)	1 (4.6%)	0.75

IOP; intraocular pressure.

## Data Availability

The data analysed in this study are available from the corresponding author on reasonable request.

## Supplementary Materials

The following are available online at https://www.mdpi.com/article/10.3390/jcm10143181/s1, Video S1: Technique of microhook ab interno trabeculotomy.
